# The relationship between the violet pigment PP-V production and intracellular ammonium level in *Penicillium purpurogenum*

**DOI:** 10.1186/s13568-016-0215-y

**Published:** 2016-07-02

**Authors:** Ryo Kojima, Teppei Arai, Hiroshi Matsufuji, Takafumi Kasumi, Taisuke Watanabe, Jun Ogihara

**Affiliations:** Depertment of Chemistry and Life Science, Nihon University, 1866 Kameino, Fujisawa, Kanagawa 252-0880 Japan; Depertment of Food Bioscience and Biotechnology, College of Bioresource Sciences, Nihon University, 1866 Kameino, Fujisawa, Kanagawa 252-0880 Japan; Graduate School of Bioresource Sciences, Nihon University, 1866 Kameino, Fujisawa, Kanagawa 252-0880 Japan

**Keywords:** Ammonium, Pigment, *Penicillium purpurogenum*, Secondary metabolism, Primary metabolism

## Abstract

*Penicillium purpurogenum* is the fungus that produces an azaphilone pigment. However, details about the pigment biosynthesis pathway are unknown. The violet pigment PP-V is the one of the main pigments biosynthesized by this fungus. This pigment contains an amino group in a pyran ring as its core structure. We focused on this pigment and examined the relationship between intracellular ammonium concentration and pigment production using glutamine as a nitrogen source. The intracellular ammonium level decreased about 1.5-fold in conditions favoring PP-V production. Moreover, *P. purpurogenum* was transferred to medium in which it commonly produces the related pigment PP-O after cultivating it in the presence or absence of glutamine to investigate whether this fungus biosynthesizes PP-V using surplus ammonium in cells. Only mycelia cultured in medium containing 10 mM glutamine produced the violet pigment, and simultaneously intracellular ammonium levels decreased under this condition. From comparisons of the amount of PP-V that was secreted with quantity of surplus intracellular ammonium, it is suggested that *P. purpurogenum* maintains ammonium homeostasis by excreting waste ammonium as PP-V.

## Introduction

The filamentous fungus *Penicillium purpurogenum* IAM15392, isolated from soil, produces PP-Y, PP-O, PP-V, and PP-R as *Monascus* pigments under specific medium conditions (Ogihara and Oishi 2002; Ogihara et al. 2000a, b, 2001). *Monascus* pigments have been used as a natural food colorant in East Asia. Recently, attention has been focused on these compounds due to their antimicrobial (Kim et al. 2006), anticancer (Zheng et al. 2010), and antioxidizing (Akihisa et al. 2005) activities. However, a nephrotoxic mycotoxin called citrinin is produced by some *Monascus* spp. (Wang et al. 2005). For this reason, in Europe and the United States, use of *Monascus* pigments is banned (Mapari et al. 2010). In contrast, *P. purpurogenum* IAM15392 is incapable of citrinin production. Hence, this fungus has the potential to become a producer of new food colorants (Dufossé et al. 2014).

We have revealed that PP-V is a monascorubramine homologue, a polyketide containing an amino group in its core structure (Ogihara et al. 2001). Polyketides are secondary metabolites produced by bacteria, fungi and plants. These compounds are widely classified into different groups such as antibiotics, cholesterol biosynthesis inhibitors, food colorants, or mycotoxins (Park et al. 2010; Manzoni and Rollini 2002; Mapari et al. 2010; Tam et al. 2015). Although nitrogen-containing compounds have biological activity (Winkler and Hertweck 2007), most polyketides are composed of polycyclic hydrocarbons. Hence, study of nitrogen-containing polyketides is interesting. We showed that the biosynthetic pathway of PP-V coincided with major *Monascus* pigments based on labeled acetate incorporation experiments (Ogihara et al. 2000a, b). However, the biosynthetic pathway of PP-V from 7-O to 7-N is not known.

Previously, we revealed that when not only inorganic nitrogen but also some amino acids are added, a violet pigment was produced, and that pigment produced on glutamine was determined as PP-V by NMR (Arai et al. 2013). Further, we showed that PP-V productivity reduced by inhibiting glutamine synthetase (GS) and that glutamine was preferred for this pigment production than glutamate (Arai et al. 2013). These results suggested that ammonium generated through glutamine degradation by glutaminase after ammonium assimilation is used for component of PP-V (Arai et al. 2013). Hence, we considered that intracellular ammonium level is important in PP-V production. In this study, we investigated the effect of intracellular ammonium level on pigment production using glutamine as nitrogen source.

## Materials and methods

### Fungal material

*Penicilliumpurpurogenum* IAM15392 was used throughout this study. Strain IAM15392 was deposited in the IAM Culture Collection, Institute of Molecular and Cellular Biosciences, the University of Tokyo, and as JCM 23216 in the Japan Collection of Microorganisms, RIKEN Bioresource Center, Japan.

### Medium and culture condition

YMA plates (10 g glucose, 5 g peptone, 3 g yeast extract, 3 g malt extract, and 20 g agar/L) were used as the stock medium for the fungal strain. One loopful of spores and mycelia of *P. purpurogenum* IAM15392 was inoculated into a 500 mL Erlenmeyer flask containing 100 mL of basal medium (20 g soluble starch, 2 g yeast extract per L of 50 mM citric acid/Na_3_ citrate buffer, pH 5.0) as culture medium for PP-O production from a stock culture on a YMA plate, and was cultivated at 30 °C, 200 rpm for 96 h.

Glutamine medium with l-glutamine (f.c. 1 or 10 mM) added to the basal medium was used to analyze the effect of glutamine on PP-V production and on intracellular ammonium level.

### Extraction of pigment and analysis

For analysis by thin layer chromatography (TLC), the culture broth was centrifuged (1600×*g*, 4 °C, 15 min). The pigment in the supernatant after filtering through No. 2 filter paper (Toyo Roshi, Tokyo, Japan) was extracted with EtOAc.

To extract intracellular pigment, mycelia were harvested after it was washed with 50 mM citric acid/Na_3_ citrate buffer, pH 5.0. The mycelia were immersed in 50 mL methanol for 24 h. This methanol extract was filtered through No. 2 filter paper and evaporated to dryness in vacuo.

Pigments were detected by TLC using a silica gel 60 plate (Merck, Darmstadt, Germany) and the developing solvent mixture *n*-BuOH:AcOH:H_2_O (12:3:5).

### In vitro reaction of PP-O with l-glutamine

*Penicillium purpurogenum* IAM15392 was cultured at 30 °C, 200 rpm for 72 h in basal medium. The culture broth was then filtered through No. 2 filter paper, and 100 mM l-glutamine (f. c. 10 mM) was added. The total 10 mL of filtrates containing PP-O and glutamine was kept at 30 °C for 10 h. The pigment that was contained in this reaction mixture was extracted with EtOAc and analyzed by silica gel TLC.

### Quantitative determination of intracellular ammonium

*Penicillium purpurogenum* IAM15392 was grown for 48, 72 or 96 h in PP-O production conditions or glutamine medium. Mycelia (0.5 g) were ground in a mortar and pestle, and 1.0 g α-alumina was added and thoroughly mixed. The 1 mL of 10 mM of phosphate buffer, pH 8.0 was added to this mixture, and then was centrifuged at 4 °C, 10,000×*g* for 20 min. Subsequently, the supernatant was recovered and centrifuged at room temperature, 10,000×*g* for 5 min after 300 μL of 0.5 M trichloroacetic acid was added. The supernatant (100 μL) was recovered and 1700 μL pure water was added. The absorbance at 450 nm (A_450_) was measured before reaction (A_1_). This mixture was left to stand for 10 min after adding 200 μL of Nessler’s reagent (Sigma-Aldrich, St. Louis, MO, USA), and the A_450_ was measured (A_2_). A_2_ − A_1_ was used to calculate intracellular ammonium levels.

### Quantitative analysis of PP-V by HPLC

The supernatant was recovered after *P. purpurogenum* IAM15392 was grown for 96 h in basal and 10 mM glutamine medium by filtering through No. 2 filter paper, and pigments was extracted with EtOAc. The extracts were used as samples. PP-V that was used as internal control was purified by the method that was indicated previously (Ogihara et al. 2000a, b). Internal control (10, 100, 1000 µg/mL) and each samples were dissolved in 0.1 or 0.05 mL of DMSO. The quantitative analysis of PP-V was performed by the reverse-phase HPLC system (Agilent HP1100 series) with a 150 × 4.6 mm i.d. X-Bridge C18 column (Waters) and a photodiode array detector (G135B DAD, Agilent Inc.). The following conditions were used:  solvent, 40 % MeCN in 0.1 % HCOOH (0–5 min), 40–80 % MeCN in 0.1 % HCOOH (5–45 min), and then 80 % MeCN in 0.1 % HCOOH (45–50 min); flow rate, 1.0 mL/min; column temperature, 40 °C.

### Replacement of medium

*Penicillium purpurogenum* IAM15392 was grown for 48 or 72 h in basal, and 1 or 10 mM glutamine medium. Then, the mycelium was transferred into fresh basal medium after washing with 50 mM citric acid/Na_3_ citrate buffer (pH 5.0), and was cultivated until the total culture time reached 96 h, including both before and after replacing the medium.

## Results

### The effect of glutamine on PP-V production

The violet pigment derived from medium to which glutamine is added was determined as PP-V (Arai et al. 2013). To verify that PP-V that detected as extracellular pigment on glutamine medium is biosynthesized in the cell, we investigated whether it was obtained from reactants of PP-O and glutamine by in vitro, and analyzed intracellular pigment by TLC.

**Fig. 1:**
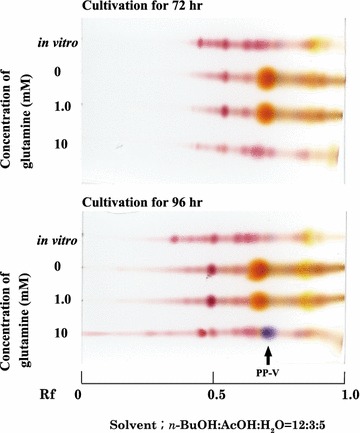
Effect of glutamine on PP-V production. *Penicillium purpurogenum* IAM15392 was grown in 500-mL Erlenmeyer flasks containing 100 mL basal medium or glutamine medium (f.c. 1 or 10 mM l-glutamine) for 72–96 h at 30 °C with shaking at 200 rpm. For TLC, the culture broth, and PP-O and glutamine reaction solution obtained in vitro were centrifuged (1600×*g*, 4 °C, 15 min). The pigment in the supernatant was extracted with EtOAc. Pigments were detected by TLC using a silica gel 60 plate with developing solvent mixture *n*-BuOH: AcOH: H_2_O (12:3:5)

The effect of glutamine on PP-V production is shown in Fig. 1. PP-V was not detected in the in vitro reaction mixture of culture filtrates containing PP-O and glutamine. Moreover, only PP-O was produced in basal medium and 1 mM glutamine medium after 72 and 96 h. In 10 mM glutamine medium, PP-V was produced after 96 h.

**Fig. 2:**
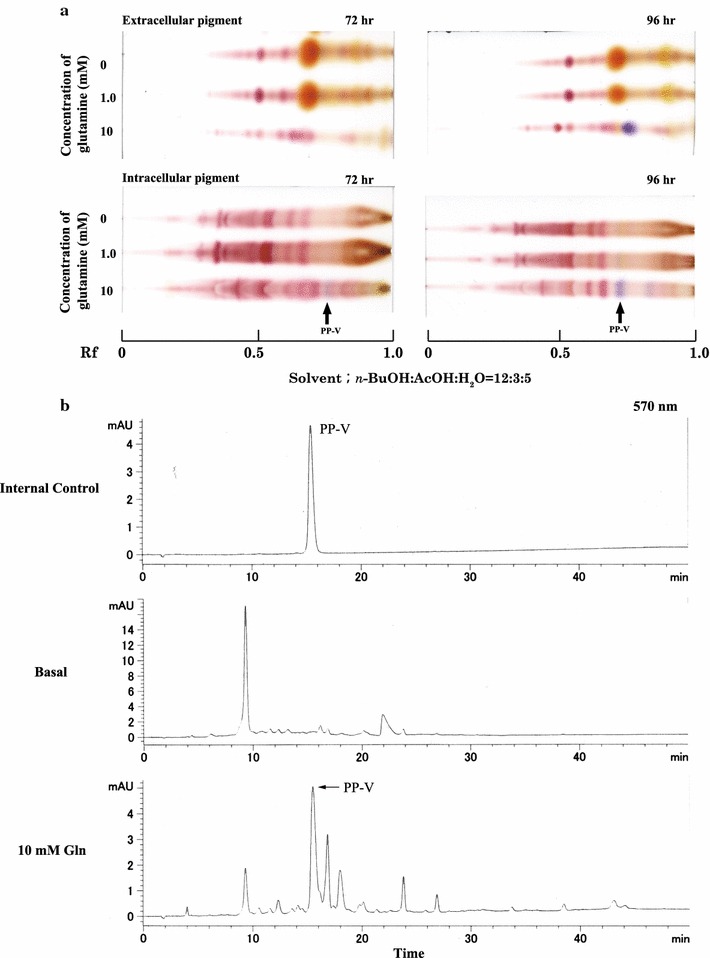
Detection of PP-V in extracts of *P. purpurogenum.* **a** TLC of the extracellular and intracellular pigment. **b** HPLC chromatogram in extracellular pigment from basal and 10 mM glutamine medium. *Penicillium purpurogenum* IAM15392 was grown in 500-mL Erlenmeyer flasks containing 100 mL of basal medium or basal medium with addition of glutamine (f.c. 1 or 10 mM l-glutamine) for 72–96 h at 30 °C with shaking at 200 rpm. The mycelia were harvested and immersed in methanol for 24 h. This methanol extract was filtered through filter paper and evaporated to dryness in vacuo. Pigments were detected by TLC using a silica gel 60 plate with the developing solvent mixture *n*-BuOH: AcOH: H_2_O (12:3:5). The extracellular PP-V was detected by HPLC (570 nm). The filtrates derived from basal and 10 mM glutamine medium for 96 h was used as samples. PP-V that was purified was used as the internal control

The results of the detection of intracellular PP-V are shown in Fig. 2a. Methanol extracts of mycelia cultured with 10 mM glutamine indicated production of a little PP-V after culturing for 72 h. The production of PP-V increased in the cultures for 96 h. On the other hand, PP-V was identified from mycelia grown in 1 mM glutamine medium. However, this pigment was not visible in the culture filtrate after growth in 1 mM glutamine medium (Figs. 1, 2a).

The chromatogram on culture broths from basal, and 10 mM glutamine medium was indicated (Fig. 2b). Extracellular PP-V was 8.52 µmol/g (cell weight) in 10 mM glutamine medium (cultivation time of 96 h).

### The measurement of intracellular ammonium level

To compare with intracellular ammonium level (*p* < 0.05) of PP-O production condition and of PP-V production condition, it was measured with Nessler’s reagent. The intracellular ammonium level of mycelia grown for 72 h increased about 1.5-fold in 10 mM glutamine compared to PP-O production conditions (0 and 1 mM glutamine) (Table 1). However, when mycelia were cultured in 10 mM glutamine for 96 h, the level decreased to almost the same as in PP-O production conditions (Table 1).

**Table 1:** The intracellular ammonium level of *P. purpurogenum* that was cultured in glutamine containing medium

Culture condition	Culture time (h)	Growth (g/100 mL)	Cell weight^a^ (g)	Ammonium level^b^ (µmol/g)^c^
Basal	72	1.84	0.50	8.37 (±0.36)^d^
	96	3.02	0.50	7.17 (±1.60)
1 mM Gln	72	1.94	0.51	8.21 (±0.94)
	96	3.06	0.48	6.52 (±0.17)
10 mM Gln	72	2.12	0.49	12.45 (±0.93)
	96	3.53	0.50	8.09 (±0.94)

^a^Cell weight was mycelia weight for this experiment

^b^This value is ammonium level of extracts

^c^This value is mole of ammonium/cell weight

^d^Values in parentheses are standard deviation (SD)

### The relationship between PP-V production and intracellular ammonium level

To examine whether PP-V utilizes waste ammonium in cell, *P. purpurogenum* IAM15392 was transferred in basal medium in which only PP-O was produced generally after cultivating with or without addition of glutamine.

**Fig. 3:**
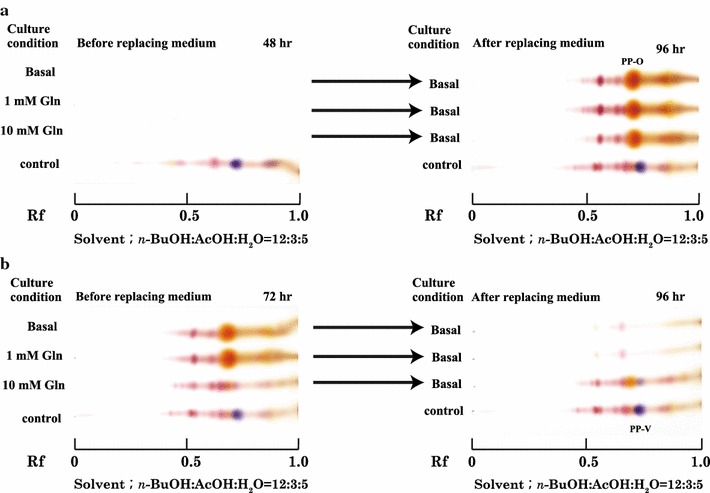
PP-V productivity of *P. purpurogenum* before and after replacing medium. **a** Pigment productivity after cultivation for 48 h and after replacing medium. **b** Pigment productivity after cultivation for 72 h and after replacing medium. *Penicillium purpurogenum* IAM15392 was grown in 500-mL Erlenmeyer flasks containing 100 mL of basal medium or 1 or 10 mM glutamine medium for 48 or 72 and transfer to fresh basal medium (no glutamine added). After then, mycelia were cultivated at 30 °C with shaking at 200 rpm for another 48 or 24 h (for a total of 96 h). The culture broth before and after replacing medium was centrifuged (1600×*g*, 4 °C, 15 min) before TLC analysis. The pigments in the supernatant were extracted with EtOAc, and were detected by TLC using a silica gel 60 plate with developing solvent mixture *n*-BuOH: AcOH: H_2_O (12:3:5). “Culture condition” describes the medium in which *P. purpurogenum* IAM15392 was grown before (*left*) and after replacing the medium (*right*): Basal represents basal medium, 1 mM Gln represents basal medium containing a final concentration of 1 mM glutamine, and 10 mM Gln represents basal medium containing a final concentration of 10 mM glutamine. PP-V derived from 10 mM glutamine medium was used as a control. The *arrow* indicates when each medium was replaced

All samples produced orange pigment when mycelia were transferred to basal medium after culturing for 48 h (Fig. 3a). In contrast, only mycelia that were transferred to basal medium after they were cultured for 72 h in 10 mM glutamine medium produced violet pigment (Fig. 3b). When the mycelia were cultivated for 72 h in basal medium or 1 mM glutamine medium, neither the violet nor the orange pigment was detected after transfer to fresh basal medium (Fig. 3b).

Table 2 shows the intracellular ammonium level (*p* < 0.05). When orange pigment was produced after mycelia were transferred to fresh basal medium, the ammonium level showed no alteration before and after replacing the medium. However, the level was substantially decreased in conditions in which the violet pigment was produced.

**Table 2:** The intracellular ammonium level of *P. purpurogenum* in before and after replacing medium

Culture condition before replacing medium	Culture time before replacing medium (h)	Cell weight (g)^a^	Ammonium level^b^ (µmol/g)^c^	Culture time after replacing medium (h)	Cell weight (g)^a^	Ammonium level^b^ (µmol/g)^c^
Basal	48	0.48	8.36 (±1.14)^d^	48	0.53	7.76 (±0.62)^d^
1 mM Gln	48	0.51	7.65 (±0.11)	48	0.53	7.38 (±0.44)
10 mM Gln	48	0.50	8.31 (±1.29)	48	0.54	6.66 (±0.67)
Basal	72	0.51	8.58 (±0.83)	24	0.50	9.28 (±0.42)
1 mM Gln	72	0.50	7.51 (±1.34)	24	0.53	9.08 (±0.53)
10 mM Gln	72	0.51	12.81 (±1.26)	24	0.53	7.55 (±0.48)

^a^Cell weight was mycelia weight for this experiment

^b^This value is ammonium level of extracts

^c^This value is mole of ammonium/cell weight

^d^Values in parentheses are standard deviation (SD)

## Discussion

First, we examined whether PP-V was produced intracellularly when glutamine was used as nitrogen source. As Figs. 1 and 2a show, the PP-V that was produced on glutamine medium was biosynthesized intracellularly.

Recently, it has been reported that secondary metabolites possess a physiological role. For example, citrinin protects cells from oxidative stress (Schmidt-Heydt et al. 2015), and ochratoxin maintains intracellular Cl homeostasis (Schmidt-Heydt et al. 2012). This suggests that pigments produced by *P. purpurogenum* may also have significance for this fungus.

PP-V is a compound containing an amino group and it is an azaphilone compound *N* derivative. Therefore, ammonium is needed to biosynthesize this pigment. Previously, we predicted that the ammonium derived from glutamine degradation is used for PP-V production **(**Arai et al. 2013).

On the basis of this consideration, we hypothesized that *P. purpurogenum* excretes unnecessary ammonium by incorporating it into the core structure of PP-V. In fact, when this pigment was produced in 10 mM glutamine medium at the point of 96 h of cultivation, the intracellular ammonium level of mycelia was reduced to as almost the same as under PP-O production conditions.

As evidence for our hypothesis, *P. purpurogenum* IAM15392 was transferred to fresh basal medium after it was cultured in medium containing glutamine and then was grown until the cultivation time reached a total of 96 h. When the first conditions were 72 h of cultivation in 10 mM glutamine medium, this fungus produced not only orange but also violet pigment in basal medium after replacing the medium. When *P. purpurogenum* was transferred to fresh basal medium after cultivating without glutamine or with 1 mM glutamine, neither PP-O nor PP-V was detectable by TLC. This suggested that the level of secondary metabolism was reduced after 72 h.

PP-V has an Rf = 0.75 (Ogihara et al. 2000a, b) and PP-O an Rf = 0.65 (Ogihara and Oishi 2002) under the TLC conditions used. Based on its Rf, the violet pigment was PP-V isolated from fresh medium after replacing 10 mM glutamine medium (Fig. 3b). Furthermore, the intracellular ammonium level after replacing medium decreased. On the other hand, the orange pigment PP-O was produced when mycelia were transferred to fresh basal medium after cultivation for 48 h (Fig. 3a). There was almost no difference between 48 and 96 h of cultivation on the intracellular ammonium level. Moreover, it is possible that this fungus produces pigments based on its growth stage.

We examined whether the amount of PP-V secreted extracellulaly corresponds with the decline in ammonium in the cell. Decrease of intracellular ammonium calculated from Table 1 was 4.36 µmol/g (cell weight). However, result of HPLC indicated that ammonium that was secreted as PP-V was higher than this at 96 h in 10 mM glutamine medium. To this reason, we might consider that PP-V biosynthesis seems promotes mostly after 72 h, and substitution of PP-O occur in the cell with intracellular ammonium that may be constantly produced from cellular nitrogen, amino acids derived from medium component in addition to added glutamine serially. On the other hand, when mycelium was cultivated another 24 h after shaking for 72 h, ammonium secreted as PP-V was 2.72 µmol/g (data not shown), but this was lower than 5.26 µmol/g (cell weight) of surplus ammonium calculated from Table 2. It suggested that a part of intracellular ammonium was used for PP-V biosynthesis by shifting metabolic system when mycelia transferred to fresh medium.

It was obvious that *P. purpurogenum* utilizes unnecessary intracellular ammonium for PP-V biosynthesis. Although ammonium generated from glutamine is utilized for biosynthesizing this pigment, it is the only one of the factor in PP-V production.

Excessive ammonium is toxic to organisms (McDermott 1957; Britto and Kronzucker 2002; Hess et al. 2006), and therefore they have machinery to detoxify it. Humans use the urea cycle to convert ammonia into nontoxic substances (McDermott 1957). *Saccharomyces cerevisiae* exports ammonium as amino acids via SPS transporters (Hess et al. 2006). However, similar machinery is unknown in fungi. It can be hypothesized that *P. purpurogenum* maintains ammonium homeostasis by incorporating an amino group into a pyran ring.

In this study, we proposed that PP-V biosynthesis is one of the mechanisms to maintain the intracellular ammonium level in *P. purpurogenum*. It is possible possibility that the intracellular ammonium level is controlled by PP-V production. In addition, it suggests relationship between nitrogen metabolism and secondary metabolism. We believe that this study contributes to elucidation of the cross-regulation between primary metabolism and secondary metabolism.
